# Research advances in forensic diatom testing

**DOI:** 10.1080/20961790.2020.1718901

**Published:** 2020-03-23

**Authors:** Yuanyuan Zhou, Yongjie Cao, Jiao Huang, Kaifei Deng, Kaijun Ma, Tianye Zhang, Liqin Chen, Ji Zhang, Ping Huang

**Affiliations:** a Shanghai Key Laboratory of Forensic Medicine, Shanghai Forensic Service Platform, Academy of Forensic Science, Ministry of Justice, Shanghai, China;; b Department of Forensic Medicine, Inner Mongolia Medical University, Huhhot, China;; c Department of Forensic Medicine, Nanjing Medical University, Nanjing, China;; d Department of Forensic Medicine, Xuzhou Medical University, Xuzhou, China;; e Institute of Forensic Science Shanghai Municipal Public Security Bureau, Shanghai, China

**Keywords:** Forensic sciences, forensic pathology, drowning, diatom, deep learning

## Abstract

In forensic practice, it is difficult to determine whether a dead body in the water resulted from drowning or from disposal after death. Diatom testing is currently an important supporting technique for the determination of death by drowning and of drowning sites, even though it is a time-consuming and laborious task. This article reviews the development of diatom testing over the decades and discusses a new method for the potential application of deep learning in diatom testing.

## Introduction

When a forensic pathologist deals with a body recovered from the water, it remains challenging to identify the cause of death, especially distinguishing drowning from other deaths [1]. A fresh drowning corpse generally has some significant signs such as pale skin and froth in the mouth and nostrils. However, these postmortem signs disappear with accelerating decomposition when a body is immersed for a long time in an aquatic environment, further making identification of the cause of death more difficult.

During drowning, liquid is aspirated into the lungs and then enters the blood circulation through the alveoli; meanwhile, some small particles in water, including sediments, microorganisms and pollen, are also carried to multiple organs and deposited in their capillaries [2]. Of these particles, a group of unicellular algae known as diatoms remain in biological tissues longer. These plankton are found in almost all aquatic and humid environments, including oceans, lakes and swamps. Because their silica shells are acid-resistant, it is easy to separate diatoms from organ tissues using acid-digestive extractions [3]. This method was firstly used in 1904 and had been widely applied. But there were many controversies [4]. The ubiquity of diatoms may cause deviations in cause of death. Meanwhile, some authors pointed out that diatoms entering into other organs were usually thought to enter from the lungs, and that it is not impossible to enter from the intestine [5]. Even so, diatom test is still one of the routine methods for diagnosing drowning.

Forensic pathologists discriminate diatom species based on visual examination of their morphologies and patterns, which are preserved well even following chemical digestion. In some cases, some species unique to certain habitats can be used as evidence to confirm a crime scene because they are not easily concealed by suspects. However, this is not encountered in most drowning cases, as most diatom species are found in different water environments. Nevertheless, diatoms are extremely sensitive to changes in environmental conditions, such as temperature and nutrients, and thus diatom species abundance varies from water to water [6]. It is helpful to determine the site where a victim falls into the water by comparing diatom species abundance patterns from tissues with those from suspect water samples.

Although diatom testing plays an important role in drowning cases, there is a need to develop more efficient methods for diatom identification and classification, as traditional diatom testing is a time-consuming and laborious task due to its low detectable rate. This article reviews the recent developments in diatom testing. We also introduce the potential of deep learning techniques to improve the efficiency of current diatom testing methods.

## Traditional chemical digestion for diatom detection

**Figure 1.**
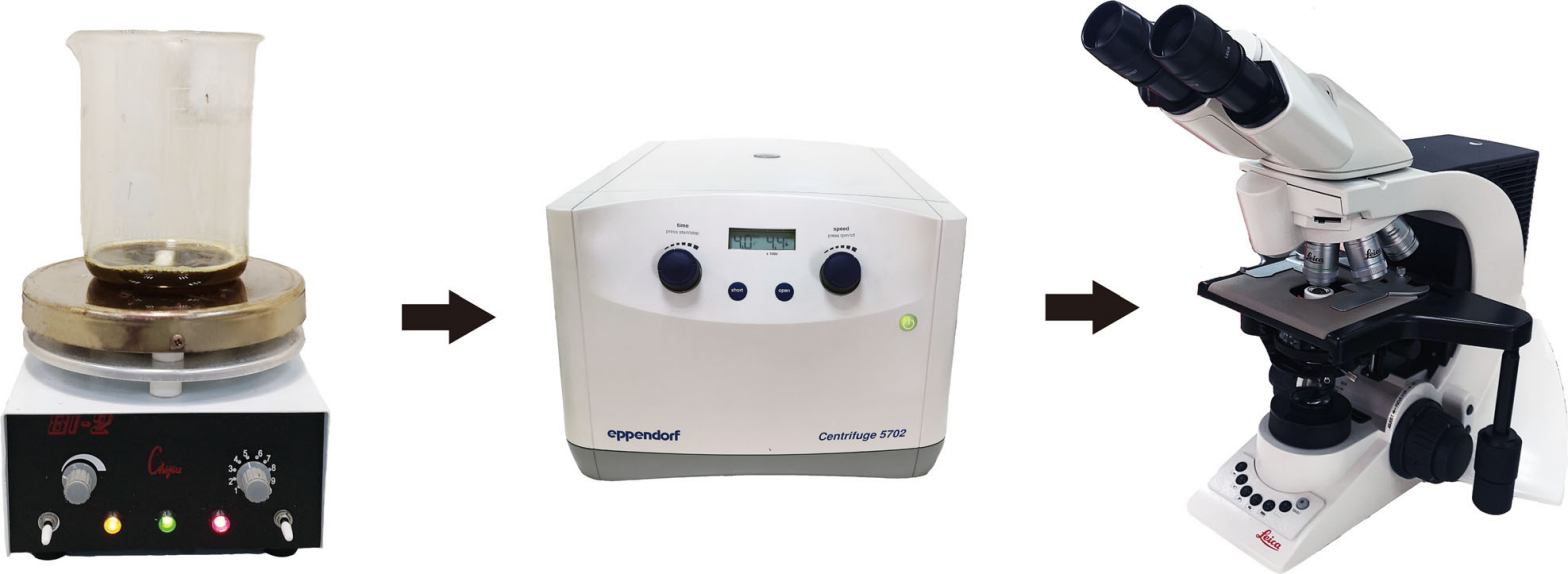
The process of traditional chemical digestion methods.

Diatom cell walls are composed of a hydrated silicon dioxide called a frustule, which is not easily destroyed under extreme conditions such as strong acid digestion. This allows overall diatom profiles to be observable when tissues are removed by chemical digestion methods. Chemical digestion methods include tissue digestion, repeated centrifugation as well as visual examination of diatoms under microscopy (Figure 1). Due to their simplicity and low cost, chemical digestion methods have been widely used in forensic casework.

**Figure 2.**
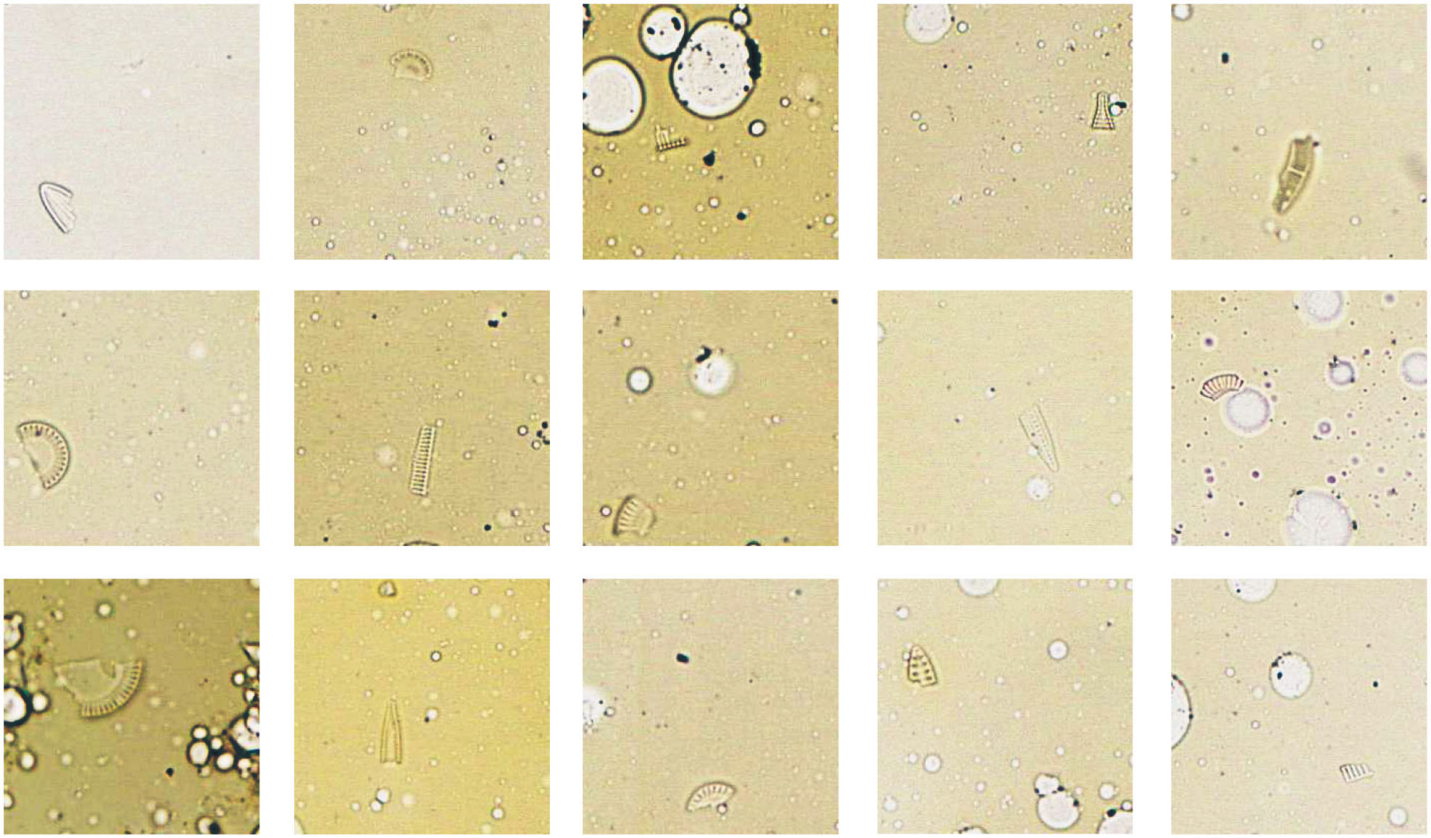
The fragments of diatom under 400 × light microscopy. Excessive digestion will cause the structure of most diatoms to be destroyed, resulting in an amount of diatom fragments [33]. Reprinted with permission.

Digestion with strong acids (e.g. nitric acid) is the most commonly used method for diatom detection. Although it is effective to eliminate organic components, the remaining waste liquid may cause serious environment pollution. Additionally, the integrity of diatom structures can be destroyed by excessive digestion (Figure 2), often leading to false-negative results.

During the past decades, there have been attempts to modify chemical digestion methods using different digestion reagents. The improvements are beneficial for separating diatoms more efficiently and environmentally from multiple organ tissues with a higher detectable rate. Comparing four chemical digestion methods, Ming *et al.* [7] found that proteinase K digestion provided a more satisfactory result with fewer samples and less pollution. However, proteinase K is too expensive to use for diatom testing with numerous samples. Subsequently, DiGiancamillo *et al.* [8] used a new method with HCl to digest pig tissues for diatom testing and compared this method to digestion methods with other reagents such as nitric acid, hydrogen peroxide and proteinase K. The use of a less corrosive acid led to the detection of more intact diatoms; this is especially useful for qualitative and quantitative analysis. It was also found that treatment with proteinase K lead to incomplete digestion, with the presence of residual organic matter that hindered microscopic observations. In 2019, Kakizaki *et al.* [9] proposed that the digestion efficiency of papain is similar to or better than proteinase K. Notably, this reagent is considerably cheaper than proteinase K, reducing experimental costs by approximately 6-fold.

## Microwave Digestion-Vacuum Filtration-Automatic Scanning Electron Microscopy (MD-VF-Auto SEM)

To date, many methods for diatom testing have only been improved by focusing on digestive reagents. Zhao *et al.* [10] developed a new method called Microwave Digestion-Vacuum Filtration-Automated Scanning Electron Microscopy (MD-VF-Auto SEM) by integrating and improving methods that had been used for diatom testing over a long period of time. In this method, microwave digestion and vacuum filtration replaced traditional acid digestion and centrifugation, respectively. The highlight of this method is Auto SEM, which could easily and efficiently identify diatoms. The SEM system automatically scans filter fields from vacuum and takes pictures of the fields for qualitative and quantitative analysis. Compared with traditional acid digestion, the MD-VF-Auto SEM method could save more time and examine more diatoms quantitatively. Subsequently, they proposed a ratio of diatom numbers in the lung tissues and drowning medium samples (the L/D ratio) to describe the possibility of identifying drowning using MD-VF-Auto SEM [11]. This study compared the number of diatoms based on the L/D ratio of a drowning group, which included 56 drowning cases, with the L/D ratio of a postmortem immersion group consisting of eight non-drowned cases. The drowning group had L/D > 1, which was higher than that of the postmortem immersion group. The result shows that the MD-VF-Auto SEM method can provide supporting evidence to identify the reason why a corpse was in water. In 2017, this team analyzed the sensitivity and detection rate of 128 drowning cases that used this method [12]. The detection rate was 100% in lung tissue samples and 97% in distant organ samples (such as the liver or kidney); these results illustrated that the MD-VF-Auto SEM method can effectively improve the diatom detection rate in a corpse. However, the MD-VF-Auto SEM method is extremely sensitive to diatom detection; therefore, there may be false-positive results due to contamination during sample collection. To solve this problem, Shen *et al.* [13] quantitatively compared diatoms from false-positive cases and real drowning cases using the MD-VF-Auto SEM method and found that the number of diatoms in non-drowning corpses and death by drowning corpses was significantly different.

## Diatom testing based on DNA sequencing

In recent years, molecular methods such as DNA sequencing have been used in diatom testing in drowning cases. DNA sequencing analyzes the base sequence of a specific DNA fragment, and has the ability to identify specific species using genetic markers. In contrast to chemical digestion methods, gene sequence-based diatom testing has higher sensitivity and is easier to implement. In addition to diatoms, other phytoplankton in the water (such as cyanobacteria and green algae) can be detected in human tissue samples from drowning cases by amplifying their specific DNA regions, which are not present in the human genome. These regions include ribosomal small subunit 16s/18s rRNA and the cytochrome c oxidase subunit I (*cox I*) gene [14–16].

Rácz *et al.* [17] applied a polymerase chain reaction (PCR)-based method to identify phytoplankton (cyanobacteria, green algae, etc.) DNA from spleen tissues during autopsy and drowning water samples. The study showed that non-diatom phytoplankton, such as small green algae and cyanobacteria, are positive in drowning cases where diatom testing was negative. This suggests that PCR detection for green algae and cyanobacteria can serve as an auxiliary for drowning diagnosis. Chen *et al.* [18] successfully classified single diatom cells using the V4 region of 18S rDNA, which can be used as a new supplementary method to conventional morphological methods. Since the accuracy rate relies on information in a database, the acquired reference data should be classified by specialists to ensure the validity of diatom testing.

## Diatom species classification

Diatom species are abundant in water and are extremely sensitive to their living environments. There are large differences in the abundance of diatom species among nearby water environments. Diatom species abundance patterns in victims’ organs may be similar to those at drowning sites [19]. Thus, accurate recognition of diatom species is useful for determining drowning sites, further providing more clues for case investigation. However, diatom classification is challenging even among people with professional knowledge in this field.

In recent years, morphology- or molecular-based methods have been proposed to classify diatom species to determine drowning sites or perform trace identification. In an animal study, Carballeira *et al.* [20] identified 86 diatom species in different waters. The similarity of the diatom species between organs and water were calculated using the Kullback-Leibler distance. It showed that there are some differences in the distribution of diatom species in different locations in the same water, suggesting that quantitative and qualitative analysis of diatoms can be used as an effective tool for determining the site of drowning. Nevertheless, diatom species recognition based on morphology is an extremely time-consuming and laborious task that requires professional expertise.

DNA barcoding is a classification method that uses specific genetic makers to identify particular species and is becoming a new method for diatom species identification. A study by Li *et al.* [21] first established a barcode for diatom morphology and diatom DNA sequences in the Yangtze River in Nanjing, China. The abundance and distribution frequency of each diatom species vary considerably among different water areas, which can support the determination of drowning sites. DNA sequencing can obtain large amounts of data in each run, and comparison of each sequence to a reference barcode library can identify the diatom classification. However, the use of DNA sequencing in forensic cases is limited due to time and cost, the complex sample preparation and processing of large-scale data. More notably, all classification methods based on DNA barcoding analysis depend to some extent on the size of the diatom database. Thus, Zhao *et al.* [22] used pyrosequencing (PSQ) to perform multidimensional analyses of diatom signals in water samples, evaluating the correlation between water samples and collection locations by multidimensional analysis of the PSQ signal curve of the diatoms in the water samples. Compared to DNA barcoding, PSQ has more accuracy and flexibility. Overall, DNA-based techniques are effective to identify more diatom species than morphology-based methods; however, expensive reagents and instruments are required for DNA sequencing, which are not available in some underdeveloped regions.

In addition to the determination of drowning sites, diatoms as trace evidence collected from clothing or shoes have been extensively used in the forensic sciences because they are not easily transferred or eliminated at a crime scene. Generally, diatoms can be extracted from their carriers, such as clothing and shoes, which are rinsed or soaked with various organic or inorganic reagents. The liquid containing diatoms is examined on standard slides using light or electron microscopy. Finally, the diatom species and their abundance are compared with suspect water samples to confirm the crime scene. Several studies have provided methodologies to extract more diatoms from different items. Scott *et al.* [23] used three methods to extract diatoms from cotton t-shirts that had been in contact with diatom-rich water and soil. They performed a correlation study on the diatom species and abundance in water samples, and investigated the effects of soaking duration on diatoms. The study supported diatom testing as an independent method for the assessment of geoforensic trace evidence and indicated H_2_O_2_ extraction is the most efficient method for the collection of diatoms from cotton t-shirts. Levin *et al.* [24] first presented an assessment study showing that diatoms on shoes could be used as trace evidence. They found that even if the shoes were in contact with water for a short period of time, diatoms would adhere to the shoes and could be detected. The study also shows that if the differences in each experimental operation can be eliminated, diatoms are highly likely to contribute to the identification of footwear traces.

## The potential of deep learning in diatom testing

Currently, traditional chemical digestion methods are widely considered as the “gold standard” for drowning diagnosis [25]. However, diatom testing with these methods remains extremely time-consuming and laborious. Indeed, only a few diatoms or their fragments, whose sizes are small or compatible with capillary endothelial junctions, reach blood circulation even though a large amount of water is aspirated into the lung tissues during drowning. The diatom loss could be further aggravated if sample preparation, such as repeated centrifugation and chemical digestion, are not handled properly. Therefore, forensic pathologists usually spend at least 50 min on diatom detection in each smear, especially for the kidney and liver. Several approaches (e.g. DNA sequencing) have shown their power for rapid diatom identification, but they are difficult to use in forensic practice due to their exorbitant cost. Some authors have proposed modified digestion methods to increase the detectable rate of diatoms, but their efficiency is not dramatically improved because diatoms have to be confirmed manually. Therefore, there is an urgent need to develop an efficient and low-cost automated diatom detection technology, specifically the automatic identification of diatom species.

Deep learning is designed to simulate human neuron function for feature extraction [26]. This technique has been a hotspot in the field of machine learning and artificial intelligence because of its excellent performance on image processing and computer vision [27]. Among deep learning algorithms, convolutional neural networks (CNNs) provide satisfactory results in image recognition, and their power can be further improved by increasing the number of training samples provided or using a special model framework called transfer learning [28]. In conjunction with digital pathology techniques, the algorithm has the ability to automatically identify pathologies on a digital slide, and therefore has been widely used in the field of biomedicine, especially tumor diagnosis and grading, including those for breast cancer [29], lung cancer [30], colorectal cancer [31] and tumor grading [32].

**Figure 3.**
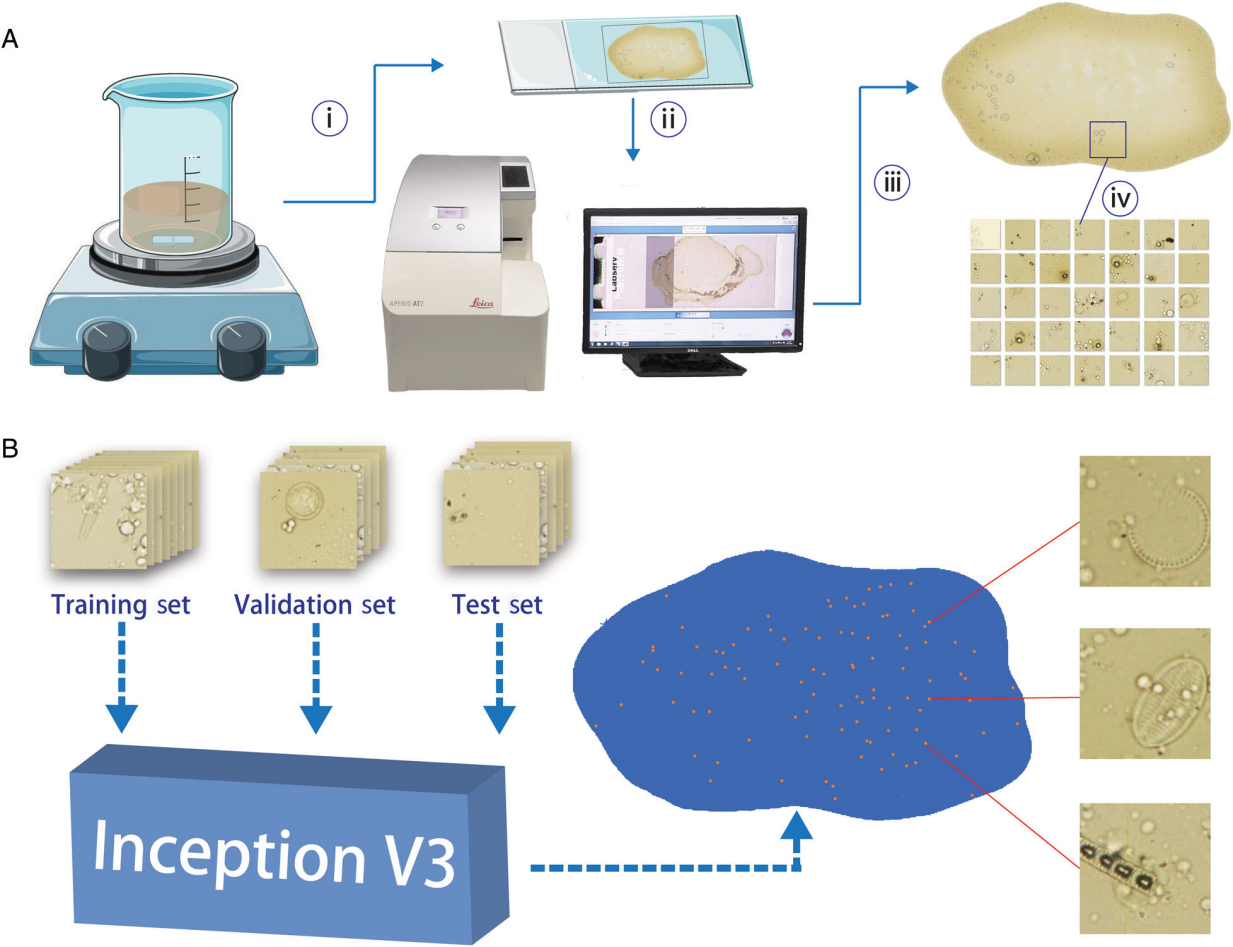
(A) The process of scanning slides. (B) In this study, the identification model was trained by the GoogleNet Inception-V3. Database was divided into training, validation and testing sets. The results were labeled into a pseudo-colour map, showing the site of diatoms [33]. Reprinted with permission.

**Figure 4.**
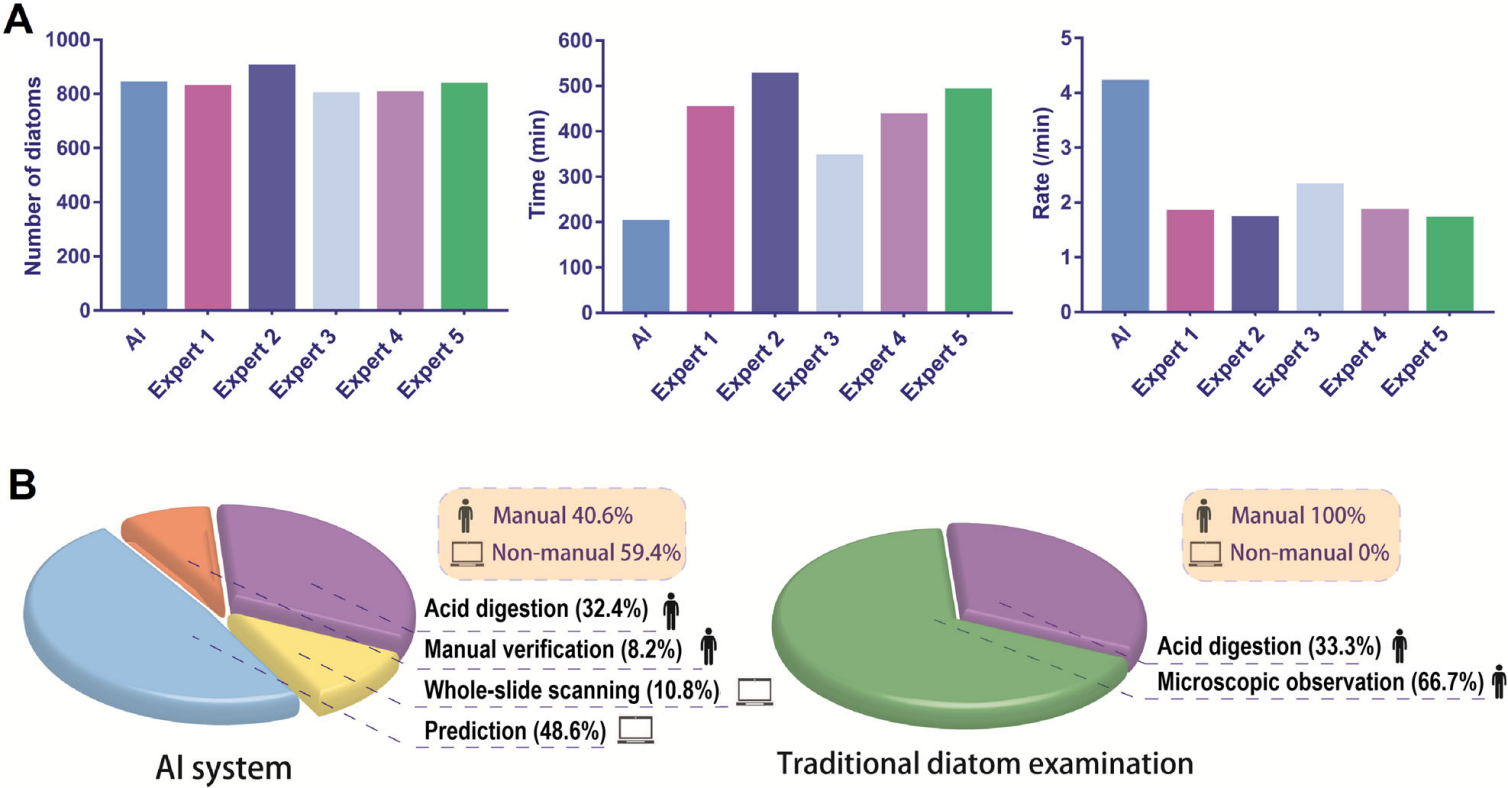
Diatom counting competition between AI system and human experts. (A) Compared to all experts, the AI system revealed greater efficiency. (B) In the whole process of diatom testing, the AI system could replace the work of manually observing and counting diatoms [33]. Reprinted with permission.

Considering its achievement in image classification, we proved that deep learning, such as CNNs, could realize automatic diatom testing [33]. Specifically, digestive tissue smears, which are similar to traditional histological slides, could also be digitized with a slide scanner (Figure 3A). Then, trained deep learning models could perform patch predictions on digital slides for diatom identification. Finally, the diatoms in tissue samples would be marked on the whole-slide (Figure 3B). In this study, the identification model achieved an accuracy of 97.67% and better performance in competition with human experts (Figure 4). The study provided an efficient method that can automatically identify diatom on the slides. Currently, only one study has been performed to classify diatom species using CNNs. Pedraza *et al.* [34] used a CNN model to successfully classify images of 80 diatom species and achieved 99% accuracy. Learned from their study, the abundance and distribution of diatom species in tissue samples can be automatically calculated based on model prediction and counting, which can then be matched to those in water samples. Moreover, since deep learning is much more efficient for collecting diatom information in different waters without any prior knowledge other than manual discrimination, a regional database associated with diatom abundance and distribution could be established within several days, which would be useful to determine drowning sites or crime scenes for case investigation.

## Conclusion

The primary task of forensics in cases involving a dead body in water is to determine whether the individual died due to drowning or whether the body was thrown into the water after death. However, due to adverse factors such as corpse decomposition and gnawing by fish or worms in the water, dead bodies in water often lack the characteristic signs of drowning, making the diagnosis of drowning extremely difficult. As common plankton in water, diatoms can enter the human body through the respiratory tract during drowning and be distributed to all organs in the body through blood circulation. Therefore, the presence of diatoms in tissues and organs can be considered a life reaction that indirectly proves that the deceased underwent a drowning process, and can be used as important evidence for the determination of death by drowning. Additionally, diatoms are extremely sensitive to the water environment in which they live. Different diatoms can breed advantageously according to their own adaptability, resulting in significant differences in the distribution of diatom species in different waters. Based on this theory, forensics can compare the abundance of diatom species in human tissue and in the water to determine the approximate drowning location in drowning cases.

Physical methods are generally difficult to implement, as instruments for implementing them are not easily attained. Moreover, treatment processes cause severe damage to diatoms, which affects the rate of diatom detection. Therefore, physical methods are rarely used in case investigations and are also not mentioned in this paper. Currently, the most common method is morphological examination based on digestion with strong acids. It is widely used in forensics laboratories because of the easily accessible and inexpensive reagents and requiring no special equipment. Additionally, the HCl digestion, proteinase K digestion and MD-VF-Auto SEM methods can preserve the integrity and improve the diatom detection rate, which is significant for qualitative analysis of diatoms. Notably, proteinase K is very sensitive for detecting diatoms in a small amount of tissue, but it is not applicable to large-scale cases due to the associated high costs. The microwave digestion device and electron microscope in the MD-VF-Auto SEM method are not common in conventional forensic laboratories. The introduction of the above methods and systems can reduce and prevent false-positive results and improve the reliability of diatom testing results. However, diatom testing based on morphological examination is often closely related to the experience of the examiner during the investigation. Therefore, developing a system based on artificial intelligence technology that can automatically identify and classify diatoms will further improve the science and objectivity of forensic diatom testing.

Although DNA-based diatom testing, which does not rely on diatom morphology, can improve the sensitivity and specificity and rapidly determine diatom species, this method relies heavily on experimental equipment and devices and has high operational requirements for the experiments. Therefore, this method is difficult to implement in basic-level forensics laboratories.

Combined with existing diatom testing techniques, qualitative and quantitative analysis of different diatom species can be performed. Dividing water bodies based on classifying diatom species and establishing the diatom species distribution in different water bodies have certain research value and application prospects in diatom testing for drowning. It can also provide useful clues and evidence for determining the site of drownings in forensic cases.

In the future, the addition of deep learning will provide automated and smarter results of diatom testing for the identification of drowning sites. These methods will more effectively serve for cases that involve a corpse found in water.
